# Six Months' Residence in England in 1836

**Published:** 1839-10

**Authors:** 


ART. V.—*Six Mois de Séjour en Angleterre, pendant l'année* 1839. Par SIRUS PIRONDI, D.M. *Marseille*, 1839.

*Six Months' Residence in England in* 1836. By SIRUS PIRONDI, M.D. —*Marseilles*, 1839, 8vo. pp. 435.

As it is always interesting, and often useful, to know what our brethren of other nations think of us and our institutions, we were induced to look into Dr. Pirondi's volume, in hopes of finding something in the journal of a physician that might be acceptable to our readers. In this we have been rather disappointed, as the author has, for the most part, reserved his medical notes with the view of making use of them in another work which he intends writing. He has, however, dropped a few observations, here and there, on medical matters, some of which we may notice. As Englishmen we may, indeed, be said to owe this courtesy to Dr. Pirondi, for surely he gives a most favorable report of us. He is full of admiration of almost all he saw, and overflowing with gratitude for the manner in which he was treated. Like most travellers who pay a mere flying visit to a foreign country, he falls into various errors and misconceptions, some rather ludicrous, particularly where he sets down some particular fact or incident as a general truth or custom. Thus he tells us that the highland regiments "are *commonly* preceded by a fine deer, which obeys the orders of the commanding officer" (p. 264);—that the young men in Oxford "study chiefly Greek and Latin,—*but especially the Bible*" (p. 131);—that the Zoological Gardens are only accessible to the "bearers of tickets from members of parliament" (p. 29);—that the fashion in Scotland is to drink their toasts after dinner "with one foot on the chair, and the other on the table," (un pied sur la chaise et l'autre sur la table, c'est ainsi qu'ils crient *hurra!*") (p. 263);—that Portobello *and Leith* are two pretty villages on the shore near Edinburgh" (p. 252); and, finally, that the very trees in England partake of the precise and formal character of the people! ("Je dirai même, sans crainte d'être démenti, que ces mêmes arbres diffèrent essentiellement de ceux du continent. Depuis leur tronc jusqu' à leurs ramifications on y voit une coupe particulière; ils tiennent encore à cette symétrie générale qu'on distingue ici partout; ils ne veulent pas détruire l'ordre grammaticale: ce sont, en un mot, des arbres Anglais.") (p. 66.) Lest, however, our Transtuedan readers should be wroth with our traveller on the score of "le toast des hommes après le diner," we must not omit to state that, while he pronounces "the hospitality, kindness, and honour" (loyauté) of the Scotch to be at least equal to the same qualities of the English; he declares "their manners to be more polished, and their ladies matchless!" (Les formes extérieurs me paraissent pourtout plus polies en Ecosse, où les femmes surtout sont d'une amabilité extrème.) (p. 262.)

Dr. Pirondi speaks most favorably of the general state and administration of our hospitals; but he complains of the patients in Guy's Hospital ("which is in itself a perfect piece of grammatical logic, never putting the adjective before the verb,") (p. 43,) being too well fed, "from the maniacal fear of seeing patients die of hunger." (p. 317.) Unless certain travellers are greatly belied, this "maniacal fear" by no means disgraces the administration of some of the hospitals in Dr. Pirondi's country.

Like all foreigners, the author is amazed at the state of organization, or rather disorganization, of the profession in this country. He confounds, it is true, the granting of degrees and licences in surgery and pharmacy with the graduation of physicians, stating that no titles can be obtained but at the great universities; and informs us that at the time he is writing "there is building in London a magnificent university" to grant all sorts of degrees; he speaks a sad and disgraceful truth, however, when he tells his countrymen that, "by a singular absurdity, no graduate can practise his art in the capital without being previously examined by one of its colleges;" and that thus "London arrogates the right of testing the medical knowledge of a doctor of medicine, although it has no power of conferring the degree." (p. 120.)

We will conclude this notice with Dr. Pirondi's account of the mode of carrying on the business at our medical societies in London, and will add an anecdote of Sir Astley Cooper, the characteristic truth of which, we are sure, no one who knows that great surgeon will call in question.

"The medical societies generally meet in the evening, and the members always arrive half an hour or more before the beginning of the business, and often take tea in an adjoining room. The president, generally armed with a small ivory hammer, opens the meeting and states the order of proceeding. The member whose turn it is rises immediately, and *reads* his paper, in the midst of profound silence, and, when he has finished, sits down. Frequently three or four minutes elapse before any one thinks of replying; but then some one gets up and delivers his sentiments. But you must not fancy that he turns to the first speaker, or rather reader: by no means—his whole discourse is addressed to the president. When he has finished his speech he seats himself, awaiting a reply. After another lapse of four or five minutes, the original orator rises and replies to the last speaker—but without ever looking at him—M. le President is the constant object of his address. In other words, there are always two advocates who seem to plead their respective causes before a judge from whom there is no appeal! This discussion at an end, silence resumes her sway once more for some minutes; but you must not from that imagine that the meeting is over: far from it—it will last two or three hours longer. One may perhaps say (adds our worthy Doctor) that this is taking things rather too coolly; but to this, again, it may be answered that there can never be too much calmness in discussion." (p. 428.)

"After the death of Dupuytren and Scarpa, medical Europe venerates in Sir Astley Cooper the first of existing surgeons. Tall, healthy-looking, robust, and broad-shouldered, he possesses one of those happy constitutions which easily supports all the labours which a superior genius can impose on it. . . . He was kind enough to make us acquainted with his researches on 'the Structure and Functions of the Thymus Gland,' with which he was then occupied; and I am the more pleased to recall this circumstance, because it enables me to record a reply of Sir Astley's, which proves delightfully the perfect truth and honesty with which he conducts his researches and experiments. While he was pointing out to us, on a most delicate preparation, the two membranes which he has found in what he calls the reservoirs of the thymus, I said to him, '*You said, and it is.*' '*No!*' he replied, '*it is, and I said.*' The scientific character of the great English surgeon breathes in this response." (pp. 320-1.)

We doubt not Sir Astley is one of those English savans "whose distinctive character," he says in another place, "is to unite a genius entirely French with a patience truly German."

